# A focus on simulation and machine learning as complementary tools for chemical space navigation

**DOI:** 10.1039/d2sc90130g

**Published:** 2022-07-11

**Authors:** Matteo Aldeghi, Connor W. Coley

**Affiliations:** a Department of Chemical Engineering, Massachusetts Institute of Technology Cambridge MA 02139 USA maldeghi@google.com ccoley@mit.edu; b Department of Electrical Engineering and Computer Science, Massachusetts Institute of Technology Cambridge MA 02139 USA

## Abstract

Computer-aided molecular design benefits from the integration of two complementary approaches: machine learning and first-principles simulation. Mohr *et al.* (B. Mohr, K. Shmilovich, I. S. Kleinwächter, D. Schneider, A. L. Ferguson and T. Bereau, *Chem. Sci.*, 2022, **13**, 4498–4511, https://pubs.rsc.org/en/content/articlelanding/2022/sc/d2sc00116k) demonstrated the discovery of a cardiolipin-selective molecule *via* the combination of coarse-grained molecular dynamics, alchemical free energy calculations, Bayesian optimization and interpretable regression to reveal design principles.

## Abstract 2

Machine learning and simulation synergistically contribute to the discovery of novel cardiolipin-selective molecules.

## Graphical abstract

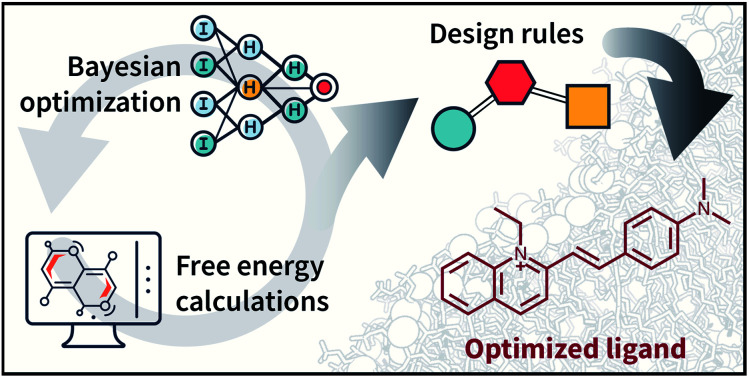

Computation is often used to accelerate the discovery of novel, functional molecules with applications from healthcare to clean energy.^1,2^ While there is a broad spectrum of roles that computation might adopt within these endeavors, a researcher is usually trying to accomplish at least one of three things: prediction, optimization, or insight. Both molecular simulations and machine learning (ML) can, in principle, be used to perform all these tasks. But the characteristics of each make them uniquely suited to some, as well as highly complementary.^3^

ML has demonstrated excellent predictive ability at low cost across a broad range of scientific domains. However, the inductive reasoning used to infer likely relationships between input (molecular structure) and output (molecular property) is fundamentally coupled to the availability of pertinent data. Simulation, on the other hand, generally follows a deductive approach in which computed properties emerge from the application of established physical laws, together with approximations required for computational tractability. Hence, prior data on a specific property is not needed when employing physics-based models for property prediction.

Molecular design can be framed as an iterative, constrained, and often multi-objective optimization problem. Data for a small number of molecules is acquired, which in turn informs the selection of the most informative subsequent candidates. Bayesian optimization^4^ is an especially suitable approach to drive molecular design given its robust, out-of-the-box performance in low data regimes. This optimization strategy relies on ML to build a cheap, surrogate model of the property being optimized, which is used to define a utility function (or “acquisition function”) that prioritizes candidates for evaluation. In principle, simulation could act as a surrogate for experimental evaluation in a Bayesian optimization framework but, given the considerable cost of molecular dynamics or density functional theory calculations, ML models are the more practical alternative. Reinforcement and active learning algorithms are a more general family of approaches that enable ML-guided decision-making for tasks including and beyond optimization.

Finally, attaining atomic or molecular-level mechanistic insight is of key interest for the understanding of the chemical and physical processes governing catalysis, molecular recognition, and self assembly. Here, simulation has the upper hand thanks to its immediate interpretability and ability to test hypotheses *in silico*. Yet, interpretable ML models can also generate insight by revealing subtle patterns hidden across multiple sets of experiments, or in the vast amount of data generated by simulations.

As demonstrated by Mohr, Shmilovich *et al.*,^5^ simulation and ML constitute flexible and complementary computational tools to achieve accurate predictions, obtain insight, drive optimization decisions, or all three.

In this collaboration, the Bereau (University of Amsterdam), Ferguson (University of Chicago), and Schneider (Johannes Gutenberg University Mainz) groups sought to discover small molecule dyes capable of selectively partitioning into cardiolipin membranes to enable the visualization and quantification of cardiolipin content. Membrane lipid composition has a profound impact on mitochondrial function, and anomalous cardiolipin content has been linked to several pathologies, from Barth syndrome to neurodegeneration. As such, cardiolipin acts as a biomarker for these conditions. Yet, the development of cardiolipin-based diagnostics has been hindered by the challenge of achieving selectivity with respect to other phospholipid membranes.

As only a handful of cardiolipin probes have been described, and have an unclear selectivity profile, Mohr *et al.* decided to use alchemical free energy calculations to compute the relative thermodynamic stability of a molecule in cardiolipin and phosphatidylglycerol membranes. These calculations rely on molecular dynamics simulations to estimate free energy differences. While their accuracy is limited by finite conformational sampling and the approximations of the molecular model, they are exact from a statistical mechanics perspective.^6^ The authors created a coarse-grained (CG) model with 6 bead types derived from the MARTINI force field,^7^ each representing sets of functional groups with different physicochemical properties, to be used in these simulations. Coarse-graining introduces the challenge of back-mapping the CG candidates to molecules, and free energy calculations with CG force fields have not been validated as extensively as atomistic ones. But it reduces the computational burden and enables a hierarchical search of chemical space.

The screening library of hypothetical coarse-grained probes comprised over 100K candidates. Given that each calculation requires 24–48 hours on a graphics processing unit, an exhaustive search is infeasible. To efficiently identify molecules with maximal cardiolipin selectivity, Mohr *et al.* thus resorted to ML-guided optimization. After running calculations for a diverse set of 100 CG molecules, they performed seven rounds of Bayesian optimization in which 60 CG molecules were evaluated in each round. A Gaussian process model was used to predict simulation output given a continuous representation of the CG candidates as input, which was obtained from a graph encoder that was part of a pretrained autoencoder model (a form of non-linear dimensionality reduction that can take a variety of inputs, including molecular graphs). Here, ML is not used to improve the selectivity estimates obtained by simulation, but to focus the free energy calculations only on the most promising and informative candidates, avoiding an exhaustive screen. With this active learning strategy, several candidates with a predicted improvement over the selectivity of the fluorescent dye 10-*N*-nonyl acridine orange of up to 184% were identified, while having evaluated only 520 (0.42% of the library).

To gain insight into what makes selective cardiolipin probes, Mohr *et al.* analyzed the results of the simulations with an easily-interpreted linear model with sparsifying L1 regularization (LASSO, a common technique for simple descriptor-based models). This approach assigned different levels of importance to the chemical patterns present in the CG library considered, highlighting groups that contributed positively or negatively toward selectivity. This analysis revealed that cardiolipin-selective molecules generally have (i) one or two positively-charged groups, (ii) a hydrophobic core, needed for alignment with or insertion into the lipid bilayer, and (iii) weakly polar groups with both hydrogen bond donor and acceptor character.

These design rules were then utilized to manually select two purchasable molecules for experimental validation with differential fluorescence anisotropy. In the future, this selection process may be automated by an inner optimization loop in which the localized area of chemical space identified *via* CG simulations is explored in full atomistic detail. One of these two molecules, quinaldine red, displayed preferential partitioning into cardiolipin-containing model membranes, validating the computational predictions and corroborating the molecular design rules inferred from the model.

Because experimental measurements for the known probe 10-*N*-nonyl acridine orange were unsuccessful, definitive evidence that the molecule discovered is more selective than previously known compounds is not available. Yet, the discovery of a cardiolipin-selective compound after the virtual screen of only a few hundred CG molecules, and the experimental testing of only two, is a testament to the power of computation in accelerating the discovery of functional molecules.

Overall, the work by Mohr *et al.* shows how simulation and ML can be used synergistically in molecular discovery. The generalizability of physics-based models and the flexibility of free energy calculations were exploited to predict a niche yet important molecular property for which experimental data is scarce. Active learning, in the form of Bayesian optimization, enabled the identification of the most promising candidates at a fraction of the cost of an exhaustive virtual screening campaign. And while more sophisticated interpretable ML approaches are available,^8^ and further validation of the design rules extracted may be warranted, the linear model used by the authors proved sufficient to drive molecular design successfully.

These techniques—alchemical free energy calculations, Bayesian optimization, and interpretable ML—are finding broad applicability in chemical design and design of experiments, even though further advances are needed to increase their accuracy and applicability in diverse chemistry research settings. Among active research areas at the interface of ML and simulations are ML interatomic potentials, which take advantage of in- or equi-variant neural network architectures and may eventually lead to more accurate free energy calculations.^9,10^ Similar supervised learning approaches are being explored for the construction of CG models, while unsupervised generative models are providing new avenues to forward- and back-map between CG and atomistic models in an automatic fashion.^11^ Finally, the development of multi-fidelity, synthetic cost- and prior knowledge-aware active learning algorithms for chemical design may enable optimal decision making tools bridging computation and experiment. With an early example of such an integrated discovery campaign, Mohr *et al.* have shown how the diversity and complementarity of the instruments present in the computer-aided design toolbox enhances the impactfulness and applicability of computation in molecular discovery.

## Author contributions

M. A. and C. W. C. wrote the manuscript.

## Conflicts of interest

There are no conflicts to declare.

## Supplementary Material
